# Medical Education Beyond Beirut: A Qualitative Study of Rural Training in Lebanon

**DOI:** 10.7759/cureus.101105

**Published:** 2026-01-08

**Authors:** Peter Kfoury, Samira Takkoush, Diane Rizkallah, Frida D Atallah, Charles E Bardawil, Ahmad A Alattar, Nayda H Bidikian, Sarah Wehbe, Salim M Adib

**Affiliations:** 1 Faculty of Medicine, American University of Beirut, Beirut, LBN; 2 Department of Epidemiology and Population Health, Faculty of Health Sciences, American University of Beirut, Beirut, LBN

**Keywords:** low- and middle-income countries, middle east countries, primary healthcare, primary healthcare centers, qualitative research, rural health services

## Abstract

Introduction: In Lebanon, a lack of systematic incorporation of rural medical training into medical curricula exacerbates healthcare disparities. This study describes the current state of medical training in rural areas and explores the needs, requirements, and limitations associated with establishing training programs in those areas.

Methods: This qualitative study used semi-structured interviews with relevant stakeholders. Purposive sampling was employed to identify key persons potentially involved in planning medical education programs and the implementation of rural primary healthcare facilities, and to query about their opinions and vision. Of 26 stakeholders invited, eight participated in the study. Inductive analysis was performed to define thematic domains from the transcribed text.

Results: Analysis identified four major themes: (i) disconnect between legal requirements and practicality, (ii) mutual benefits, (iii) reality of rural medical training: existing efforts and challenges, and (iv) a practical model for implementation. Responses covered the status of rural medical training in Lebanon, rural communities’ needs, challenges, and a proposed implementation model. Despite legal requirements mandating rural service for practicing physicians in Lebanon after graduation, enforcement is inconsistent, with medical training and practice concentrated in urban centers. Barriers such as supervisor shortages and financial constraints hinder any rural implementation. Collaborative efforts between local and national governments and academic institutions are recommended to address these challenges through new legislative frameworks, quality improvement of primary healthcare centers, and targeted funding.

Conclusions: Future initiatives should address existing obstacles to establish a sustainable and equitable healthcare system nationwide. While limited by a small sample size and the qualitative scope, this research can serve as a foundation to create a model for improving medical education and healthcare delivery in rural areas, in Lebanon, the Arab region, and other low- and middle-income countries facing similar challenges.

## Introduction

Access to adequate healthcare services in rural areas remains a global challenge, with significant implications for public health outcomes [1-3]. The disparity in physician distribution between rural and urban areas persists in numerous countries worldwide, prompting concerted efforts from medical education programs and governmental bodies to address this issue [4,5]. These disparities are reported in many Arab countries, including but not limited to Egypt, the United Arab Emirates, and Morocco [6,7]. Published data from Arab countries remain relatively limited, and much of the existing literature focuses on workforce distribution, without proposing alternative approaches to tackle these inequalities.

Strategies aiming at bridging this gap fall into three categories: a utilitarian approach, which offers financial rewards as well as other benefits to physicians as an incentive to practice in rural areas; a coercive approach, which involves the deployment of physicians to practice in underserved areas for a certain period after graduation; and a normative approach, which attracts physicians to work in rural areas through education and awareness campaigns [8]. Within the normative approach, efforts to bolster the rural medical workforce encompass initiatives such as recruiting students with rural origins and fostering positive exposures to rural health during undergraduate education [9]. Studies suggest that early exposure to rural medical training (i.e., during medical school) is correlated with physician retention in rural areas [10,11].

Despite Lebanon’s high physician per capita ratio, healthcare professionals remain disproportionately clustered in urban areas, with 67% of rural primary health care centers (PHCCs) lacking the necessary medical personnel to operate effectively [12]. This uneven distribution may stem from declining interest in primary care and rising sub-specialization rates [13], further limiting rural access and contributing to poorer health outcomes, including higher mortality rates and increased lifestyle risk factors [14].

Despite the recognized benefits of early exposure to rural medicine for medical trainees and the rural community at large, there is currently no policy to systematically incorporate such experiences into the medical training or practice in Lebanon. There is limited research on the role of rural medical training in Lebanon, particularly concerning its benefits for the rural population. The predominant focus on urban clinical training raises questions about the lack of exposure to rural medicine in Lebanese medical curricula.

Given these gaps, a qualitative study design was selected as the most appropriate approach to explore stakeholder perceptions, identify context-specific barriers, and generate informed insights in an area where quantitative data are limited. Accordingly, the objectives of this study are to: (i) describe the present landscape of medical training in rural areas in Lebanon; (ii) explore stakeholder perceptions regarding the needs and potential benefits of rural medical training; (iii) identify perceived barriers and limitations to the implementation of rural training programs; and (iv) examine proposed models and policy-relevant strategies for integrating rural medical training into the Lebanese medical education system.

## Materials and methods

Study design

This article reports the results of a qualitative study based on a review of relevant documents and a series of semi-structured interviews with key stakeholders regarding their perceptions of medical training in rural areas within Lebanon.

Participants’ recruitment

Purposive sampling was employed to identify key stakeholders based on their experience and knowledge of medical training and practice in rural areas. The research team identified 26 stakeholders from the public sector, such as current or previous members of the Health Committee at the Lebanese Parliament (MPs), the Ministry of Public Health (MOPH), as well as primary health care (PHC) practitioners, deans, program directors, and clerkship directors at academic institutions, and family medicine (FM) specialists.

Although the sample size was small, participants were selected to reflect a range of institutional roles and perspectives relevant to rural medical training. This approach aimed to mitigate potential selection bias by ensuring diversity in professional background and decision-making authority. Stakeholders were invited to participate through email to reduce any sense of obligation, and those who expressed interest were then sought out to schedule a convenient time for an interview.

Study tool: a semi-structured questionnaire

The interviews were guided by a semi-structured questionnaire with open-ended “core” questions developed based on our literature review [12,15,16] and some aspects of interest for our objectives. In general, core questions addressed the status, challenges, and potential benefits of designing and/or incorporating medical practice training programs for rural PHCCs in Lebanon, and the legal, logistical, and organizational aspects of such programs. Additional questions were tailored to specific interviewees according to their respective roles and experience (Appendix).

Study procedures

Stakeholders’ participation in interviews was voluntary, and participants had the option to withdraw at any time. Consent was obtained from interviewees regarding audio recording and note-taking. Participants were assured that their identity would not be publicly disclosed. The interviews were conducted in English and Arabic, with all audio recordings transcribed into English. Interviews were conducted face-to-face at a place selected by the interviewee or virtually through Zoom™. No repeat interview was carried out.

Data analysis

Qualitative analysis was performed using an inductive thematic approach using the transcribed text. Transcripts were reviewed to identify initial codes, which were then grouped into broader thematic categories based on patterns of similarity and divergence across interviews. Coding and theme development were conducted through repeated review of the original audio recording and the transcripts. To enhance analytic rigor, triangulation was employed through the involvement of multiple members of the research team in the review and interpretation of the data.

Data saturation was considered achieved when no new themes or conceptual insights emerged from successive interviews, and when subsequent data largely reinforced previously identified themes. Given the focused research question and the high level of stakeholder expertise, thematic saturation was reached within the final interviews.

Ethics

This study did not require Institutional Review Board approval, as instructed by the American University of Beirut (AUB), because it only includes interview procedures. All data have been anonymized, and interviewees provided approval for the publication of their transcripts without their identities being disclosed.

## Results

Eight stakeholders participated in the survey, including: three academic leaders in medical education (AL1-3), two senior family medicine specialists (FM1-2); one primary health expert from the Ministry of Public Health (PHC1); one previous member of the Lebanese Parliament (MP1), himself a GP from a rural area; and one current member of the Lebanese Parliament’s Health Committee (MP2). Five of the eight participants were female (63%), and all had at least 10 years of professional experience in medical education, healthcare delivery, and/or rural health policy.

Four main themes emerged from the analysis (Table 1). Beyond describing existing gaps, stakeholders offered interpretive insights into the structural and policy-related factors perpetuating the urban concentration of medical training. Participants consistently framed rural workforce shortages not merely as a logistical issue, but as a consequence of outdated regulatory frameworks, limited institutional incentives, and the absence of formalized rural training pathways within medical curricula. These perspectives suggest that rural training deficiencies are embedded within broader governance and educational systems rather than arising from individual physician preferences alone.

**Table 1:** Themes and subthemes This table outlines the major themes and associated subthemes relevant to rural medical training. It covers foundational aspects, adaptations needed for rural settings, challenges to sustainability, and practical strategies for implementing effective rural medical education programs.

Theme	Subtheme
Disconnect between Legal Requirements and Practicality	The law on rural medical service
	Refusal of the coercive approach
	Re-evaluating the definition of rural areas in Lebanon
Mutual Benefits	Educational benefits and clinical intuition
	Impact on local communities
Reality of Rural Medical Training: Existing Efforts and Challenges	Limited rural training
	Barriers to academic supervision
	Financial limitations and resource allocation
A Practical Model for Implementation	Mandatory clinical rotation in medical school curricula
	Preparation of primary health care centers

Theme 1: disconnect between legal requirements and practicality

The Law on Rural Medical Service

A 1962 law (article number 10823, volume 42, page 1662-1663, October 9th, 1962) mandated a two-year medical service in rural areas before final licensure to freely practice in Lebanon [17]. The decree exempted female Lebanese physicians from this obligation, as well as specialty physicians who present proof of their specialization. It also outlined specific geographical criteria as rural areas, defining them as any region in Lebanon outside of Beirut and its suburbs, the larger cities and towns on the Mediterranean coastline (Tripoli, Sidon/Saida, Byblos/Jbail, and Tyr/Sour) and the largest two cities in the inner eastern Bekaa Valley (Zahle and Baalbek).

Refusal of the Coercive Approach

From the time of enactment, the law was applied inconsistently. Following the start of the protracted Lebanese civil wars (1975-1991), which fractured the country along sectarian lines, medical services became limited to select governmental and university hospitals within Beirut. Following the return of civil peace in 1991, disagreements emerged over the reinstatement of the two-year requirement among physicians and parliamentarians. Rural medical training was not restored, and the bulk of general and specialized training became largely centered around the Greater Beirut (GB) area. Nearly all stakeholders took a strong stance against implementing a mandatory two-year rural medical training after graduation.

‘This mandate contradicts the freedoms guaranteed by the Lebanese constitution. The Lebanese state can only implement such a law if it provides the necessary accommodations, transportation, and resources. Since there is no investment in these areas, it cannot impose such a mandate.’ (AL1)

Re-evaluating the Definition of Rural Areas in Lebanon

The participants agreed that the rural-urban classification outlined in the 1962 law is outdated and does not reflect Lebanon’s current demographic and infrastructural realities. Instead, participants emphasized functional and contextual criteria over administrative definitions, which aligns with those outlined by the Food and Agriculture Organization of the United Nations [18].

‘Defining rural areas in Lebanon is tricky. Relying solely on population size is inaccurate, given the lack of reliable population data and the large number of refugees from neighboring countries residing in traditionally rural regions, such as the Beqaa Valley in the east. A more accurate approach is to classify rural areas based on land use. Rural areas can be defined as regions where agriculture and animal husbandry are the primary sources of livelihood and economic activity. Using this criterion, rural areas in Lebanon are primarily concentrated in Akkar in the North, the Beqaa Valley in the East, and South Lebanon.’ (AL2)

**Figure 1:**
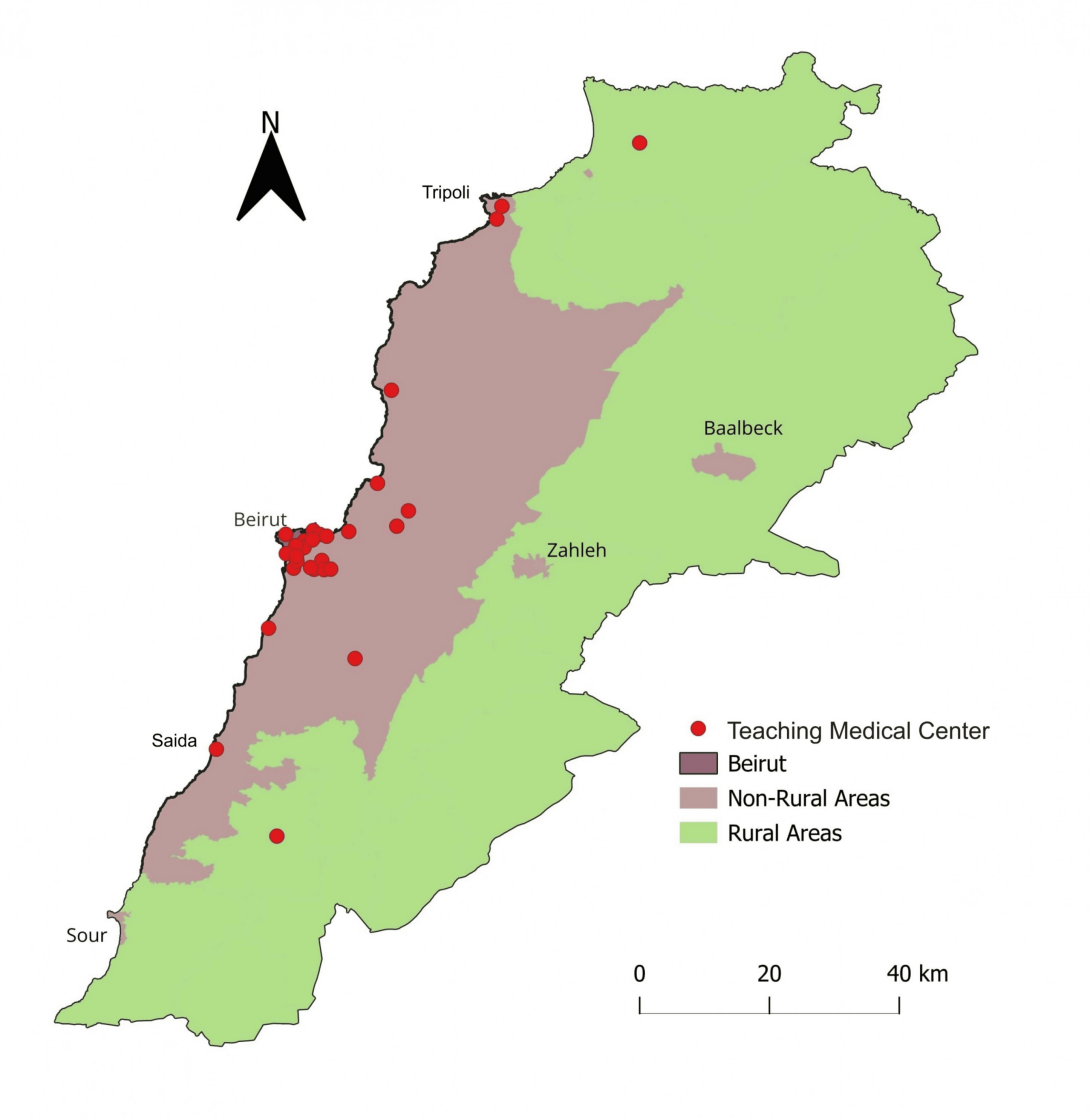
Map of Lebanon highlighting rural areas based on land use Rural areas (colored in green) were defined as regions where agriculture and animal husbandry are the primary sources of livelihood, including Akkar in the North, the Beqaa Valley, and South Lebanon. Teaching medical centers pinpointed on the map represent the teaching hospitals and/or medical centers that host medical students from the eight medical schools in Lebanon. Source: DIVA GIS [19], Processing: QGIS, Credit: Peter Kfoury.

Others suggested that any area within an hour’s commute to a major city should not be considered rural. This criterion aligns with regions where agriculture remains the primary source of livelihood. Based on this consensus, Figure 1 presents a clearer and more up-to-date map delineating rural regions in Lebanon.

Theme 2: mutual benefits

Educational Benefits and Clinical Intuition

Most interviewees emphasized the educational benefits of rural training, including hands-on experience, diverse case exposure, and cultural immersion. In rural areas, medical students are expected to actively participate in patient care under the guidance of experienced senior practitioners, with limited access to resources. This necessitates a greater reliance on clinical intuition and experience, rather than on the availability of diagnostic technologies.

Rural rotations provide exposure to a wide array of medical cases that they may rarely encounter in urban or tertiary care settings. It also offers greater exposure to primary care that permits students to manage common conditions (e.g., delivery, diabetes…), and screen for cancers such as breast cancer. With every such encounter, students are challenged to apply their medical knowledge and adapt their diagnostic and therapeutic approaches to different clinical scenarios.

Some interviewees expressed that the core benefit of rural training lies not in the rural location itself, but in the experience of working in any underserved environment.

‘It remains questionable whether rural training is inherently beneficial to medical students, or if the true value lies in gaining experience in any underserved, low-resource setting. Whether in rural areas or the slums of cities, these underserved environments provide invaluable learning opportunities, as students may one day work in similar settings.’ (AL3)

Impact on Local Communities

The absence of satisfactory healthcare in a rural community is an important contributing factor to the desire to leave, either to the city or even abroad. By providing training opportunities for medical professionals in rural settings, medical schools can help alleviate shortages in manpower and foster the development of a skilled healthcare workforce that is better equipped to meet the diverse needs of rural populations. The establishment of affiliations between academic medical centers and rural healthcare facilities strengthens the integration of rural practice into the broader medical community.

‘It would bring forth a practice of medicine that is closer to the standards of evidence-based practice. It can reinforce the skills of doctors and nurses in the area. It can also improve the medical awareness of the people through awareness campaigns and gatherings.’ (MP1)

A notable example was the AUB’s partnership with “Ain w Zain Hospital” (2000-2006), where a one-week clerkship provided exposure to conditions and practices as close to rural settings as possible. AUB faculty participated in teaching rounds, and an entire ward was dedicated to students. During this time, the hospital’s medical records were updated, and overall standards improved significantly, culminating in the hospital receiving an award for “Best Rural Hospital in Lebanon”.

Rural programs can mitigate the financial burden on populations by reducing their reliance on tertiary care centers in urban areas. Patients would no longer need to incur the expenses associated with delayed care and having to travel to distant urban centers and stay there for non-tertiary healthcare needs. This localized approach also fosters a sense of community engagement and empowerment, as rural residents and visiting medical trainees become active participants in the local microeconomy.

‘The economy in those areas would improve tremendously if a large number of students relocated there. Guest houses would be built, and the area will be prepared to welcome trainees. The economy would improve with the formation of suitable infrastructure.’ (MP2)

Theme 3: reality of rural medical training: existing efforts and challenges

Limited Rural Training

Several medical schools have established affiliations with hospitals in areas within and outside the capital. Most of these training sites are located within GB, with only a few hospitals situated outside the capital in small towns that are not considered rural. Only two universities send a limited number of students to rural areas, as defined in Figure 1. Beirut Arab University (BAU) places students at El Youssef Hospital in the northern district of Akkar, while the Lebanese University - the only public medical school in Lebanon - sends a select group of students to the Governmental Hospital in Nabatiyeh in the southern part of the country.

Opportunities for medical students to train in PHCCs are expanding, supported by the Ministry of Public Health; most recently, AUB’s Department of Family Medicine began sending students on day-trips to a PHCC in Damour, a semi-rural coastal town 30 minutes away from GB, to broaden their exposure to non-urban healthcare settings.

Barriers to Academic Supervision

In rural areas, the shortage of healthcare professionals extends to attending physicians who are essential for supervising and mentoring medical trainees. Without adequate supervision, students may not receive the guidance necessary for their education, thus compromising the quality of their training and patient care. Attending physicians in rural settings may already be overburdened with patient care responsibilities, leaving limited time and capacity for mentoring students.

Financial Limitations and Resource Allocation

Students participating in rural training programs may face financial challenges, particularly related to transportation and housing. The need for temporary housing accommodation, if available at all, further adds to the financial strain on students or their sponsoring institutions. To overcome such limitations, stakeholders proposed implementing a funding program for trainees and rural healthcare facilities, funded by the central government, local municipalities and/or university training centers.

Theme 4: a practical model for implementation

Mandatory Clinical Rotations in Medical School Curricula

While key stakeholders had different opinions relating to the best approach to implement a rural training program, most pointed out that, if deemed necessary, it should be incorporated as part of the medical school curriculum in the last year before graduation.

The feasibility of enacting this legislation either on a national scale or on a decentralized, institutional scale is subject to ongoing deliberation among stakeholders. There is a recognition of the necessity for extensive dialogue and collaborative efforts to reach a consensus on the most effective implementation strategy. This includes considerations such as the pooling of resources of different medical schools, as well as broader policy implications for medical education, healthcare delivery, and overall developmental plans nationwide.

Preparation of PHCCs

The capacity of PHCCs needed to host medical students and the availability of the necessary mentoring and facilities can be incorporated in the MOPH accreditation standards and become the primary blueprint towards the establishment of a rural training program. By enhancing the existing infrastructure and providing more resources to PHCCs, the MOPH and medical schools facilitate meaningful experiences for trainees while simultaneously strengthening primary healthcare services in these communities.

‘We have 271 PHCCs with an infrastructure capable of hosting medical trainees in rural areas. They can accommodate medical trainees in the final years of their training. There would be a standardized model to host and train students all over Lebanon. Not only would it be possible, but also necessary: there is a need for physicians and trainees in those centers. Teaching programs can be standardized by all medical schools under this one PHCC network.’ (PHC1)

## Discussion

This study provides a contextual analysis of rural medical training in Lebanon, highlighting its potential benefits and the structural and logistical challenges associated with its implementation. While PHCCs represent promising platforms for rural clinical rotations, significant barriers remain related to supervision, infrastructure, and sustained engagement.

The legal mandate for rural service, established in the 1960s, has become obsolete and is not currently enforced. Stakeholders emphasized that reinstating a compulsory rural service nationwide would face constitutional, logistical, and ethical challenges. International evidence suggests that early exposure to rural practice positively influences health professionals’ decisions to pursue careers in rural areas [10,11,20]. Thus, a normative approach would be more favorable than a coercive approach.

Implementing rural rotations could improve access to preventive and primary care and contribute to broader developmental goals. These include stimulating local economies, reducing healthcare-related expenditures for rural populations, and mitigating urban migration by enhancing rural livability and employment opportunities. Stakeholders also emphasized the educational value of rural training for medical students. There was a consensus in suggesting rotations of at least two months in rural settings to be incorporated in the last medical year before graduation with a medical degree. Such training would provide opportunities to develop clinical acumen in low-resource settings and improve cultural competence and sensitivity through interactions with diverse populations. These experiences can foster patient-centered care, reduce reliance on unnecessary diagnostics, and promote adaptability, skills applicable across all clinical settings [21,22]. 

However, such interventions alone are insufficient to correct the persistent imbalance of specialists between rural and urban areas in Lebanon and comparable contexts [12,23]. The success of rural training initiatives also depends on strong community-academic partnerships. While much of the supporting evidence originates from high-income countries like Australia [24], Canada [24,25], or Germany [10], the applicability of these strategies in low- and middle-income countries, including Lebanon, remains underexplored. Stakeholders expressed confidence that similar collaborations could strengthen Lebanon’s rural health system and would address both healthcare and workforce development needs. Nevertheless, structural challenges, including political instability, limited and inconsistent funding, and governance constraints, may further complicate nationwide implementation and should be considered in program planning.

The implementation of training programs can be further facilitated by utilizing existing resources, such as PHCCs and dormitories. A phased implementation strategy was also suggested; it would begin with rotations in urban areas outside Beirut, such as Baalbek, Saida, or Zahle, followed by a gradual expansion to less-equipped rural towns. While most PHCCs, distributed across Lebanon, adhere to regulations and MOPH accreditation standards [12], their current infrastructure may not fully accommodate medical trainees. Infrastructural gaps are relatively easier to address compared to the more complex need for providing effective supervision of medical training for students and/or residents in PHCCs. To overcome this challenge, stakeholders proposed providing skills improvement and rehabilitation, as well as financial incentives for physicians who may already be available in a rural area, but whose medical training falls short of the supervision level.

Another strategy is exemplified by the Ministry of Education in Nepal. The Ministry initiated a program that grants the top 10% of every class in medical school a full scholarship covering their education, in exchange for a two-year rural service commitment. A survey conducted in 2009 found that these students were more inclined to work in rural areas compared to their peers [26]. A similar approach could be piloted in Lebanon, particularly for publicly funded medical graduates. 

To strengthen the educational value of rural training despite low patient volumes, clinical rotations could expand beyond the scope of curative care. Activities such as home visits, school health programs, preventive services, and community health education can provide meaningful learning opportunities while enhancing public health impact. This broader approach ensures continuous student engagement, even in low-volume settings, and aligns with the role of PHCCs as community-based hubs. It also fosters a deeper understanding of population health and equips trainees with the skills needed for comprehensive, community-oriented medical practice.

This study has several limitations. While data saturation was achieved, the sample size remained small (eight stakeholders), and perspectives from students, residents, and community members were not included, which may limit the generalizability of findings. Purposive sampling may introduce selection bias, although participants were chosen to represent diverse institutional roles. Additionally, recommendations are exploratory and should be interpreted as preliminary guidance rather than definitive policy directives. 

## Conclusions

While the literature on rural health in general, and medical training in rural areas in particular, remains scarce and perhaps outdated in Lebanon and the Middle East, we are hoping that this study can elicit a national dialogue with larger representatives of other concerned groups, such as medical students and residents, and local governments and municipalities. Collaborative pilot-based training programs in rural regions, supported by targeted funding and public-private partnerships, could serve as a foundation for sustainable models of rural medical education. Integrating rural rotations and community-based clinical experiences, with clear evaluation metrics, into medical curricula will be essential to ensure effectiveness and scalability.

Future research should focus on mixed-methods and longitudinal evaluations to assess the impact of pilot programs on physician retention, rural healthcare access, and community health outcomes. Comparative studies across the Middle East may further inform best practices and policy development. 

## Appendices

Topic guide questionnaire

General Questions

1.    What would you consider rural vs. urban areas in Lebanon?

2.    Based on your knowledge, is there any formal medical training - whether optional or mandatory - currently taking place in rural primary healthcare centers? If yes:

                    i.            How is this training being implemented?

                   ii.            How does it compare to training experiences in the past?

                 iii.            Some university programs send trainees to “underserved” areas. Which schools conduct such initiatives? Would you consider these areas “rural”?

3.      What benefits do the students and communities draw from such initiatives?

4.      Can we apply similar initiatives to rural areas?

5.    What is your perspective on the involvement of residents and medical students in medical training at rural primary health care centers (PHCCs)?

A.      If you believe it is important:

              i.            What are the minimum requirements that should be available to trainees in these PHCCs? (e.g., in terms of facilitators, human resources, quality control, the presence of mentors, residents vs. trained attendings, online vs paper records)

             ii.            What are the advantages of integrating trainees into rural health centers?

           iii.            How could this benefit the primary healthcare centers?

           iv.            What are the drawbacks of such programs?

             v.            How can this negatively impact medical training?

           vi.            How could they negatively impact PHCCs? (e.g., burden, cost, resources…)

         vii.            What are the barriers to implementing these programs? (e.g., Practicality and logistics/accreditations/Lebanese laws)

B.      If you believe it does not provide added value: Many countries (e.g., Australia and Canada) have integrated primary healthcare training programs that serve rural healthcare needs and provide a good training experience.

6.      Do you think implementing similar programs in Lebanon would be possible? Beneficial?

7.      What are the barriers to adopting such models in Lebanon? (e.g., resource limitations, infrastructure gaps, legislation, cultural differences)

            i.            Should medical training be restricted to tertiary care centers?

           ii.            Do you see value in expanding training to PHCCs?

           iii.            What impact would such programs have on Lebanon’s healthcare system?

Specific Questions

1.    Since PHCCs are considered developmental projects, how could medical education within these centers contribute to their long-term developmental goals?

2.    Conversely, in what ways could integrating medical training programs into PHCCs hinder their development?

3.    Legislation of medical training at primary healthcare centers:

                    i.            An MD by law was required to practice medicine for the first 2 years in rural areas. When was this law legislated, and how was it implemented?

                   ii.            Another law mandated primary care providers to complete a residency before practicing medicine, with special training provided to physicians who have been practicing for a while. Do you think that these laws are relevant today

4.    How prepared are the primary health care centers to accommodate medical training?

5.            A law was passed that organizes primary healthcare centers in Lebanon.

                 i.            Were medical trainees considered part of the organizational power in PHCCs?

                ii.            How can the involvement of medical trainees in PHCCs affect the organization of these centers (pros and cons)?

6.    University programs: There are 7-8 universities in Lebanon that have MD programs.

                i.      Are there any plans to extend medical training outside university hospitals, particularly outside Beirut and its suburbs?

                ii.      What are the requirements that should be provided by the primary health care centers to offer a learning experience that is comparable to hospital training?

                iii.      What are the main concerns that could impede such training rotations?

                iv.      How do the existing laws and university accreditations affect such plans?
